# Effects of proteasome inhibitor MG-132 on the parasite *Schistosoma mansoni*

**DOI:** 10.1371/journal.pone.0184192

**Published:** 2017-09-12

**Authors:** Enyara R. Morais, Katia C. Oliveira, Renato G. de Paula, Alice M. M. Ornelas, Érika B. C. Moreira, Fernanda Rafacho Badoco, Lizandra G. Magalhães, Sergio Verjovski-Almeida, Vanderlei Rodrigues

**Affiliations:** 1 Departamento de Bioquímica e Imunologia, Faculdade de Medicina de Ribeirão Preto, Universidade de São Paulo, Ribeirão Preto, SP, Brasil; 2 Departamento de Bioquímica, Instituto de Química, Universidade de São Paulo, São Paulo, SP, Brasil; 3 Centro de Parasitologia e Micologia, Núcleo de Enteroparasitas, Instituto Adolfo Lutz, São Paulo, SP, Brasil; 4 Grupo de Pesquisa em Produtos Naturais, Núcleo de Pesquisa em Ciências Exatas e Tecnológicas, Universidade de Franca, Franca, SP, Brasil; 5 Laboratório de Expressão Gênica em Eucariotos, Instituto Butantan, São Paulo, SP, Brasil; George Washington University School of Medicine and Health Sciences, UNITED STATES

## Abstract

Proteasome is a proteolytic complex responsible for intracellular protein turnover in eukaryotes, archaea and in some actinobacteria species. Previous work has demonstrated that in *Schistosoma mansoni* parasites, the proteasome inhibitor MG-132 affects parasite development. However, the molecular targets affected by MG-132 in *S*. *mansoni* are not entirely known. Here, we used expression microarrays to measure the genome-wide changes in gene expression of *S*. *mansoni* adult worms exposed in vitro to MG-132, followed by *in silico* functional analyses of the affected genes using Ingenuity Pathway Analysis (IPA). Scanning electron microscopy was used to document changes in the parasites’ tegument. We identified 1,919 genes with a statistically significant (q-value ≤ 0.025) differential expression in parasites treated for 24 h with MG-132, when compared with control. Of these, a total of 1,130 genes were up-regulated and 790 genes were down-regulated. A functional gene interaction network comprised of MG-132 and its target genes, known from the literature to be affected by the compound in humans, was identified here as affected by MG-132. While MG-132 activated the expression of the 26S proteasome genes, it also decreased the expression of 19S chaperones assembly, 20S proteasome maturation, ubiquitin-like NEDD8 and its partner cullin-3 ubiquitin ligase genes. Interestingly, genes that encode proteins related to potassium ion binding, integral membrane component, ATPase and potassium channel activities were significantly down-regulated, whereas genes encoding proteins related to actin binding and microtubule motor activity were significantly up-regulated. MG-132 caused important changes in the worm tegument; peeling, outbreaks and swelling in the tegument tubercles could be observed, which is consistent with interference on the ionic homeostasis in *S*. *mansoni*. Finally, we showed the down-regulation of Bax pro-apoptotic gene, as well as up-regulation of two apoptosis inhibitor genes, IAP1 and BRE1, and in contrast, down-regulation of Apaf-1 apoptotic activator, thus suggesting that apoptosis is deregulated in *S*. *mansoni* exposed to MG-132. A considerable insight has been gained concerning the potential of MG-132 as a gene expression modulator, and overall the data suggest that the proteasome might be an important molecular target for the design of new drugs against schistosomiasis.

## Introduction

According to the World Health Organization, schistosomiasis is an acute and chronic parasitic disease, which affects over 258 million individuals. The transmission has been documented in 78 countries, of which 52 are at higher risk [1,2]. Praziquantel (PZQ) is the only drug currently recommended by WHO for preventive chemotherapy programs [3,4]. It is safe, mostly available, administered in one oral dose and inexpensive [4]. However, the existence of less susceptible strains, reduced cure rate, and treatment failure after successive PZQ doses reinforce the need for novel strategies of control of this parasitosis, based on safe and effective treatments [5,6]. The identification and use of synthetic and biological inhibitors of the proteolytic activity has mainly contributed to the characterization of essential functions of the 26S proteasome in various processes and metabolic pathways in eukaryotic cells [7,8]. The effect of proteasome inhibitors, such as MG-132 (*N*^α^-benzyloxycarbonyl-l-leucyl-l-leucyl-leucinal), lactacystin and other compounds, has been described in *Entamoeba histolytica*, *Entamoeba invadens* [9], *Leishmania mexicana* [10], *Trypanosoma cruzi* [11], *T*. *brucei* [12], *Plasmodium berguei* [13], *Toxoplasma gondii* [14] and *Plasmodium falciparum* [15,16], and for treatment of leishmaniasis, Chagas disease and sleeping sickness [17]. Besides, proteasome inhibition has been described as a promising tool to control a range of other diseases [18–21]. In 2005, Guerra-Sá et al. [22] showed that MG-132 acted on the proteasome system and caused accumulation of high molecular weight ubiquitinated proteins, also being able to reduce the number of lung stage schistosomula, the worm burden and consequently, the egg output in experimental schistosomiasis in mice [22]. In schistosomiasis, eggs are the main cause of pathology, and the observed MG-132 effect of decreasing the egg output [22] argues for the importance of further characterizing the molecular targets affected by MG-132 in *S*. *mansoni*.

The information generated through the genome and transcriptome projects opened up new perspectives for understanding the parasite biology at the molecular level, in addition to the identification of genes encoding proteins that might be promising candidates for vaccines and potential targets for new drugs [23–29].

As a post-genomic tool, the microarray technology has produced large-scale transcriptional data in *Schistosoma* [30–34]. These studies have provided lists of genes potentially involved in the development and sexual differentiation of the parasite, which may contribute to a better comprehension of these processes and identification of new pathways as targets for possible therapeutic intervention [30–32,35–41]. More recently, the RNA-seq approach has contributed additional valuable information about the parasite transcriptome [38,42,43]. It is apparent that genome-wide gene expression analyses should be useful to assess the impact of drugs like MG-132 proteasome inhibitor on the parasite. In the current study, we investigated the effect of MG-132 treatment on *S*. *mansoni in vitro*, using large-scale gene expression assays. The present findings contribute to expand our knowledge about the parasite proteasome biology and to identify target molecules that might be important in the design of new drugs for prevention and control of schistosomiasis.

## Material and methods

### Ethics statement

All procedures involving care and handling of animals were reviewed and approved by the Ethical Committee for Animal Care of University of São Paulo (protocol number 021/2009), in accordance with national (Brazilian legislation, CEUA, 11.794/2008) and international principles for laboratory animal welfare. Six-week-old female BALB/c mice from the Animal House of the University of São Paulo, Brazil, weighing 20±2.5 g, were used. The animals were housed in groups of fifteen in wire-bottomed cages and maintained in a room with controlled conditions (22 ± 2°C, 55% humidity, and 12 h light/dark cycles), and food and filtered water were provided *ad libitum* for 8 weeks. The cages were sanitized and animals were monitored twice a week. The criteria used to assess animal health and well-being were the observation of the behavior of the mice when opening a cage and the general physical state was evaluated by observation of the color of the foot pads (anemia detection) and hydration by the eye and facial fur appearance [44]. Mortality during the experiment period was not observed. *S*. *mansoni* adult worms were recovered from the hepatic portal system and the liver of mice, 8 weeks after infection, by perfusion under anesthesia with an intra-peritoneal injection of 40 mg/kg sodium thiopental.

### Chemicals

The proteasome inhibitor MG-132 (*N*^α^-benzyloxycarbonyl-l-leucyl-l-leucyl-leucinal) was purchased from Sigma-Aldrich (St. Louis, MO, USA) and dissolved in DMSO.

### *In vitro* treatment of *S*. *mansoni* with MG-132

The Luis Evangelista (LE) strain of *S*. *mansoni* was used. The life cycle of the parasite was routinely maintained by passage through *Biomphalaria glabrata* snails from a colony that was kept at the animal house of the University of São Paulo. Cercariae shed from infected snails were used for infecting BALB/c mice at the animal house (approximately 70 to 100 cercariae per animal) through the transcutaneous route [45]. *S*. *mansoni* adult worms were recovered from the hepatic portal system and the liver of mice, 8 weeks after infection, by perfusion with citrate saline (0.85% sodium chloride; 1.5% sodium citrate) that was pumped through a perfusing needle placed in the left ventricle of the heart [45]; the perfusate and worms were collected through the hepatic portal vein that was slit open [45]. These parasites were then incubated for 24 h (37°C, 5% CO_2_) in RPMI 1640 medium (Invitrogen, Life Technologies Inc., Carlsbad, CA, USA) supplemented with penicillin (100 UI/mL), streptomycin (100 μg/mL), and 10% bovine fetal serum (Gibco, ThermoFisher Scientific Inc., Wilmington, DE, USA), containing either 50 μM MG-132 (treated group) or 1% DMSO (vehicle, control group). This concentration of DMSO has been shown by our group [46] not to affect parasite mortality and parasite motility, and by others [47] not to affect parasite worm morphology. It has been previously observed that upon treatment of the parasites with 50 μM MG-132, there was no death of the worms, however there was a decrease in oviposition in mice infected with treated parasites [22]. Here, the colorimetric quantification test based on 3-(4,5-dimethylthiazol-2-yl)-2,5-diphenyl tetrazolium bromide (MTT) [46] was used to monitor treated parasites viability. Each adult worm couple was placed in a separate well of a 24-well plate, 50 μM MG-132 or vehicle was added and the couples were observed until they were separated; after 24 h, approximately 80% of couples had separated in the treatment assays and no separation was observed in the controls. In the treatment assays, only separated couples were further used, either for the viability test or for the measurement of gene expression with microarrays. For the viability test, the MTT assay reagents were added to each couple 24 h after the addition of MG-132 or vehicle, and six technical replicate couples were measured for each condition. Three biological replicates were performed on different days, and the mean ± S.D. was calculated. For the microarray experiments, four biological replicates were obtained for each experimental condition (treated or control). For each treatment replicate, 5 batches containing 20 separated worm pairs each were pooled. For the controls, the same number of paired couples was used.

### Total RNA isolation and microarray experiments

Total RNA was extracted from adult worms (treated and control groups) using Trizol reagent (Invitrogen), according to the manufacturer’s protocol. The RNA samples were then treated with DNAse I (QIAGEN, Hilden, Germany) and purified using Qiagen RNeasy mini kit (QIAGEN). The integrity of RNA samples was evaluated through microfluidic electrophoresis in the Bioanalyzer equipment (Agilent Technologies, Santa Clara, CA, USA). RNA concentration was measured in the NanoDropTM 1000 spectrophotometer (ThermoFisher Scientific Inc., Wilmington, DE, USA). Gene expression analysis was performed using the 4 x 44K oligoarray platform, an oligonucleotide microarray slide containing 39,343 probes representing *S*. *mansoni* gene fragments, designed by Verjovski-Almeida et al. [34], and manufactured by Agilent Technologies. The platform probe annotation is available at the Gene Expression Omnibus (GEO, https://www.ncbi.nlm.nih.gov/geo/) under the accession number GPL8606.

For each biological replicate, 500 ng of total RNA was amplified and fluorescently labeled using Agilent Quick Amp Labeling Kit (Agilent Technologies), following the manufacturer’s protocol for linear amplification and labeling of poly-A RNA by T7-RNA polymerase. For each biological replicate a total of 825 ng Cy3- and Cy5-labeled cRNA from treated *vs* control samples, respectively, was used for hybridization in each array. A second technical replicate was obtained for each biological replicate, by labeling with opposite dyes, in a two-color dye-swap approach. The slides were washed and processed according to the Two-Color Microarray-Based Gene Expression Analysis (Quick Amp Labeling) Protocol (Agilent Technologies) and scanned on a GenePix 4000B scanner (Molecular Devices, Sunnyvale, CA, USA). Data were extracted using Feature Extraction software (Agilent Technologies). Raw data is available under accession number GSE57722 at GEO (https://www.ncbi.nlm.nih.gov/geo/).

### Analysis of microarray data

Only genes with significantly detectable signal in at least 75% of all replicates in each group (treated or control) were analyzed (using the IsPosAndSig column information from Feature Extraction data output). The intensities were normalized by LOWESS algorithm [48], and the log _2_ ratios between treated and control groups were calculated. Pearson’s correlation coefficient (*r*) of signal intensity was used to evaluate biological and technical variability across the samples. For the treated group: three biological replicates (*r* = 0.92, range = 0.85–0.97) and technical replicate (*r* = 0.98, range = 0.97–0.99). For the control group: three biological replicates (*r* = 0.94, range = 0.91–0.99) and technical replicate (*r* = 0.98, range = 0.97–0.99).

We used Significance Analysis of Microarray (SAM) [49] as the statistical test, to identify differentially expressed genes using the log2 (treated/control) ratios. We performed SAM one-class analyses and the genes were considered significantly differentially expressed at q-value ≤ 0.025. Hierarchical clustering of selected genes was generated using Spotfire Decision Site software (TIBCO Software Inc., Palo Alto, CA, USA). For a gene represented in the array by multiple probes, we selected a single representative probe with the lowest coefficient of variation (obtained from intensity data of previous replicated experiments with the 44K oligoarray).

Functional analyses of differentially expressed genes were performed using the Ingenuity Pathway Analysis tool (IPA, QIAGEN Bioinformatics, QIAGEN, Redwood City, CA, USA). For this purpose, we annotated *S*. *mansoni* genes encoding putative homologs to human proteins; the putative homolog should have similarity with a BlastX e-value lower than 10^−10^ and coverage of at least 60% of the human homolog. The RefSeq number of each human homolog was associated to each *S*. *mansoni* gene and the expression data was uploaded to the IPA System version 7.6. We included all gene/protein relationships described in the IPA database as experimentally observed and/or predicted with high confidence in the model animals, namely human, mouse and rat.

### Real-time quantitative PCR (q-PCR) validation of microarray data

One μg of DNAse-treated total RNA was reverse transcribed using the ThermoScriptRT-PCR System (Invitrogen), in the presence of oligodT primer and Reverse Transcriptase, following the manufacturer's instructions. The resulting cDNA (0.5 μl) was used as template in the real-time qPCR using SYBR green PCR Master Mix (Applied Biosystems, ThermoFisher Scientific Inc., Wilmington, DE, USA), according to the manufacturer’s protocol. The qPCRs were run in an ABI 7500 Real-Time PCR System (Applied Biosystems). The experiment was performed with three independent biological replicates. Nuclease-free water was used as a non-template control (NTC), and three technical replicates were run for each biological replicate. The relative levels of transcripts were calculated using the comparative ΔΔCt method, and α-tubulin (GenBank accession M80214) was used as the endogenous control. Student t-test was used to calculate the statistical significance between samples; the observed expression differences were considered to be statistically significant at *p* < 0.05. Gene specific primers were designed using the Primer3 software (http://primer3.sourceforge.net/) with default parameters (**S1 Table**).

### Scanning electron microscopy

Tegument morphology of the parasites exposed to MG-132 treatment was investigated by scanning electron microscopy. After exposing the parasites to 50 μM MG-132 or to vehicle (control), the worms were rinsed in PBS (stabilized at 37°C to prevent heat shock), fixed in 3% glutaraldehyde for 60 min at 37°C, incubated for 60 min at room temperature, washed twice and maintained in PBS at 4°C. The parasites were then post-fixed in 1% OsO_4_ in 100 mM sodium phosphate buffer, pH 7.2, for 2 h at 4°C. After post-fixing, the material was washed in 100 mM PBS three times, dehydrated in increasing concentrations of ethanol, and then maintained at the critical point (Critical Point Dryer 030, Bal-Tec, Balzers, Liechtenstein). Finally, the dried specimens were mounted on aluminum stubs, and the material was visualized through scanning electron microscopy using a JSM 6610LV electron microscope (JEOL Ltd., Tokyo, Japan) operated at 25 kV.

## Results

### Gene expression profile of *S*. *mansoni* adult worms treated with MG-132

We have previously observed that treatment of *S*. *mansoni* parasites with 50 μM MG-132 caused no death of the worms, however there was a decrease in oviposition in mice infected with treated parasites [22]. Here we treated paired adult worm couples with 50 μM MG-132 or with vehicle and observed that after 24 h, approximately 80% of couples had separated in the treatment assays and no separation was observed in the controls. For further assays, both the viability test and the gene expression measurements, we only used the males and females that had been separated upon treatment, and compared with paired couples from the controls. We used the MTT colorimetric quantification test [46] to monitor treated parasites viability, and found that upon treatment with MG-132 for 24 h there was a statistically significant 30% decrease in the viability of separated adult worms, but no death of the parasites (S1 Fig).

**Fig 1:**
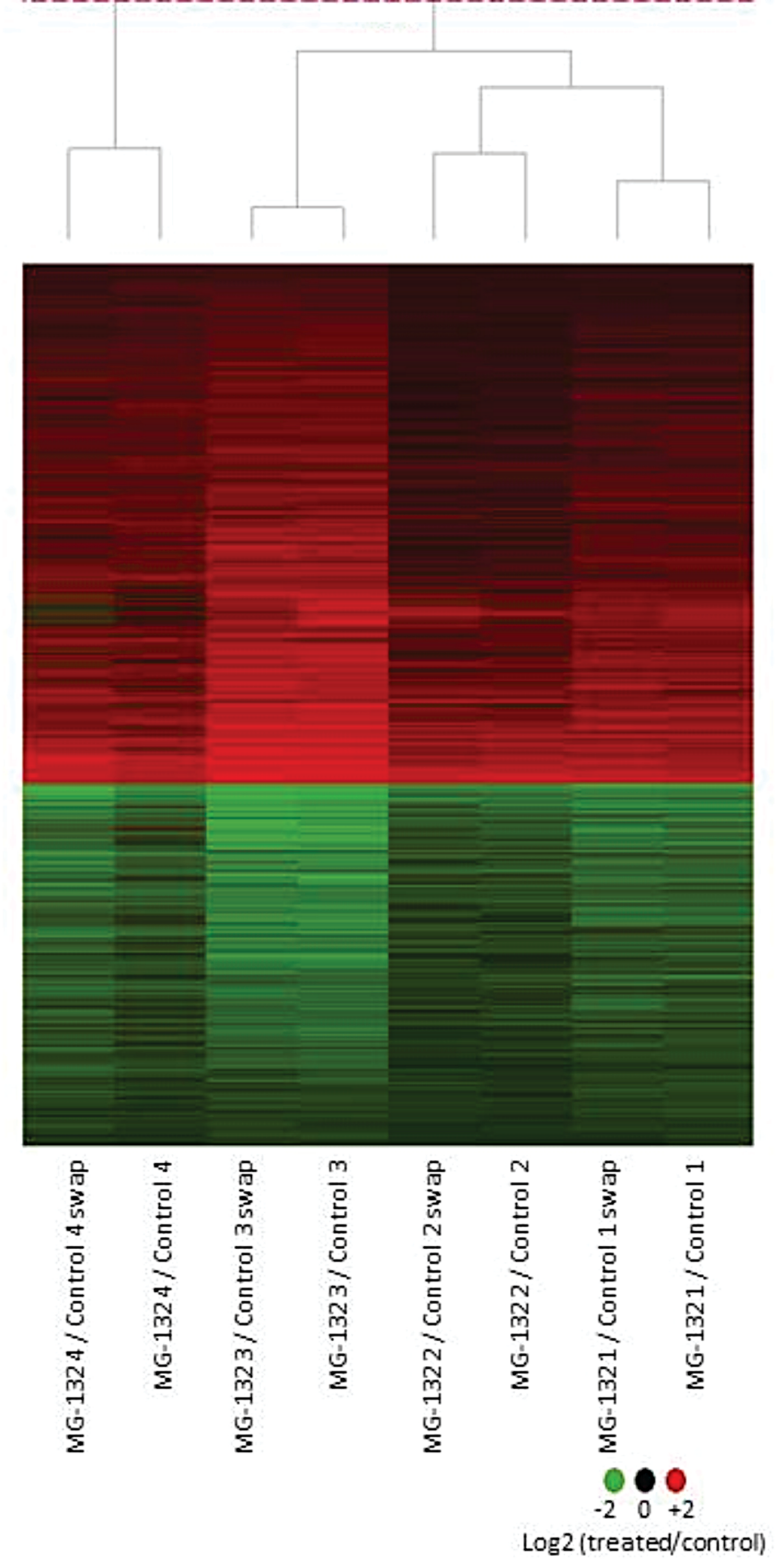
Effect of MG-132 on gene expression profile in *Schistosoma mansoni* adult worms. Adult worm pairs were treated for 24 h with 50 μM MG-132. Microarrays were used to measure gene expression on a large scale. The figure shows a group of 1,919 genes with a statistically significant (*q-value* ≤ 0.025) differential expression in adult worms treated with MG-132 *versus* controls. Each horizontal line represents a gene and each column represents an experimental replicate. There are two technical replicates for each one of four biological replicates. Genes with transcription induced by treatment are shown in red, genes with repressed transcription are in green, and the color intensity is proportional to the log2 ratio (treated/control), as indicated by the color scale at the bottom.

Large-scale gene expression of MG-132-treated and control adult worm parasites was measured with custom-designed microarrays [34], and 10,056 out of 19,907 genes and gene fragments probed on the arrays were detected as expressed (Table 1). Of these, a total of 1,919 genes exhibited a statistically significant (*q-value* ≤ 0.025) differential expression in *S*. *mansoni* adult worms exposed for 24 h to MG-132 compared with controls (Fig 1). About 64% of the differentially expressed genes, i.e. 1,231 genes are Smp-annotated genes predicted on the *S*. *mansoni* genome, while the remaining 36% belong to other categories (Table 1).

**Table 1:** Number of genes with expression detected in each category generated by the analysis of microarray experiments.

Category	Expressed genes	MG-132 differentially expressed genes (*q-value* <0.025)	MG-132 up-regulated genes	MG-132 down-regulated genes
			(*q-value* <0.025)	(*q-value* <0.025)
*S*. *mansoni* predicted genes^a^	3730	722	429	293
Anti-sense genes to *S*. *mansoni* predicted genes	2604	510	298	212
*S*. *mansoni* homologs to *S*. *japonicum* predicted genes	354	75	47	28
Anti-sense genes to *S*. *japonicum* predicted genes	258	48	27	21
Homolog genes on GenBank^b^	858	159	95	64
Anti-sense genes to homolog genes on GenBank	553	90	53	37
No match genes (with no homologs)	1,699	315	181	134
**TOTAL**	**10,056**	**1,919**	**1,130**	**789**

^a^ *S*. *mansoni* Smp genes predicted in the parasite genome [23].

^b^ Genes not predicted in the *S*. *mansoni* genome annotation, however annotated as having homologs in other species, using *S*. *mansoni* ESTs sequence comparisons with BLASTp against the GenBank database of genes from all species [34].

### Functional analysis of differentially expressed genes

To enhance our understanding of the molecular mechanisms by which *S*. *mansoni* responds to the stress caused by MG-132, we performed functional analyses of the differentially expressed genes using the the IPA tool. These functional analyses provide the biological context for gene expression changes by integrating available literature information on model organisms (human, mouse, rat) regarding molecular and chemical interactions, cellular phenotypes as well as about signaling and metabolic pathways.

The IPA analysis revealed all interactions previously described in the literature between MG-132 and the *S*. *mansoni* differentially expressed genes for which there are homologs in the model organisms (S2 Fig). Among all 1,919 *S*. *mansoni* differentially expressed genes there are 376 genes that have homology to the human counterparts, out of which 287 were modeled by IPA into a single network of interacting genes (S2 Fig) known in the literature to be primarily or indirectly related with the effect of MG-132 in these model organisms.

**Fig 2:**
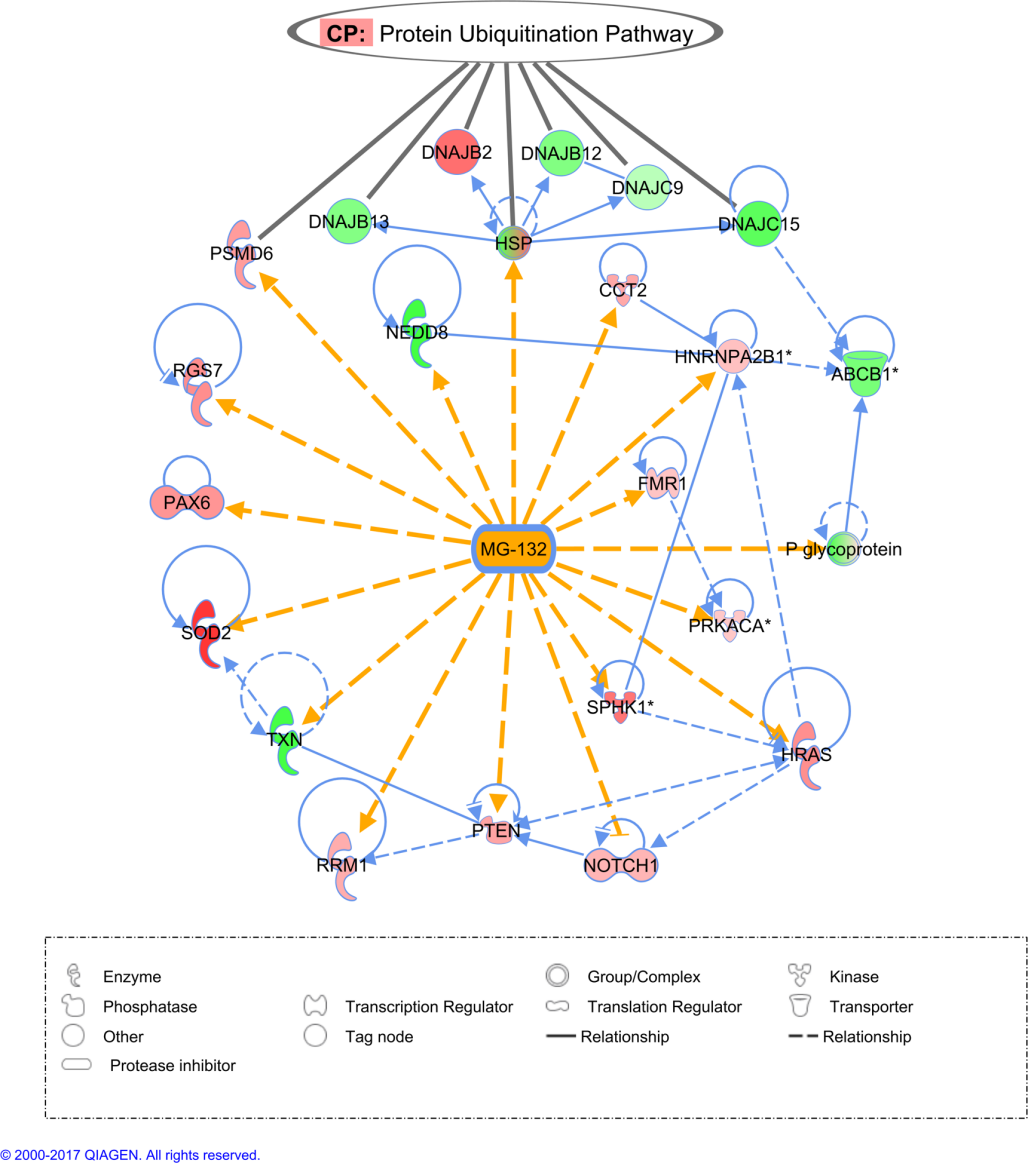
Highlighted gene interaction network between MG-132 and *S*. *mansoni* differentially expressed genes whose homologs in model organisms are described in the literature as being affected by the compound. Each line represents a direct (filled line) or an indirect (dashed line) relationship between the gene products. Yellow lines highlight the interactions between compound MG-132 and the affected gene products according to the literature; blue lines indicate the interactions among the differentially expressed genes. An arrowhead indicates a change in expression, activation or localization of the gene product, and an arrow line with a broken end indicates an inhibitory relationship. Each symbol shape represents a different gene category, as indicated in the symbol shapes legend at the bottom of the figure. Gene names are given inside the symbols, and the corresponding Smp gene annotation for each *S*. *mansoni* gene is given in S2 Table. Red color indicates that the expression of the indicated gene was up-regulated, and green indicates that the expression was down-regulated in *S*. *mansoni* adult worms treated with MG-132 compared with the control. This interaction network was obtained with the Ingenuity Pathway Analysis (IPA) tool as previously described [50,51], and the analysis is further detailed in the Methods. At the top of the figure, genes belonging to the canonical pathway (CP) called “Protein Ubiquitination Pathway” are pointed with black lines.

As part of the above network, there were 17 genes or gene complexes whose expression was affected by MG-132 in *S*. *mansoni*, for which the human homologs are described in the literature as primarily affected by the compound. These genes are highlighted in Fig 2, and the S2 Table gives the name of the corresponding *S*. *mansoni* homolog genes in this network and summarizes the effect of MG-132 described in the literature.

### Differentially expressed genes in adult worms treated with MG-132 are involved in particularly interesting functions

We highlight a selection of 30 significantly (q ≤ 0.03) down-regulated genes (Fold change < 0.73, or at least 1.3 X down-regulation) and 30 significantly (q ≤ 0.03) up-regulated genes (Fold change > 1.3) with interesting biological functions in adult worms treated with MG-132 compared with the control (Tables 2 and 3, respectively). These tables highlight the molecules that may be involved in the disruption of parasite homeostasis. The genes are particularly interesting because they are involved in signal transduction processes, protein ubiquitination and degradation, and apoptosis, among other functions. Regarding apoptosis, Bax gene, encoding a key pro-apoptotic protein, was down-regulated (Table 2), whereas Apoptotic protease activating factor 1 (Apaf-1), encoding another pro-apoptotic protein, was up-regulated (Table 3). S1 and S2 Datasets list all the 1,919 genes with repressed or induced expression, respectively, in adult worms treated with MG-132.

**Table 2:** Representative list of 30 repressed genes in *S*. *mansoni* adult worms in response to MG-132 treatment.

ProbeName	Contig	Gene Smp	Gene annotation	Fold Change
Q2_P08728	C902198.1	Smp_171620	methylthioadenosine phosphorylase, putative	0.17
Q2_P32288	C908635.1	Smp_087230	5-formyltetrahydrofolate cyclo-ligase, putative	0.17
Q2_P17855	C921267.1	Smp_120510	steroid dehydrogenase, putative	0.18
Q2_P21581	C805071.1	Smp_002160	DNA methyltransferase 1 associated protein 1 [Homo sapiens]	0.2
Q2_P40316	JAP03944.S	Smp_095190	apoptosis regulator bax, putative	0.23
Q2_P13201	C910061.1	Smp_137460	cytoplasmic polyadenylation element binding protein (cpeb), putative	0.24
Q2_P22707	C806602.1	-	smooth muscle myosin heavy chain 11 isoform SM2A [Homo sapiens]	0.24
Q2_P38626	JAP09001.C	-	tumor protein p63 regulated 1-like [Homo sapiens]	0.26
Q2_P09722	C903654.1	Smp_159440	schwannomin interacting protein, putative	0.27
Q2_P11632	C906993.1	Smp_135770	zinc finger protein, putative	0.27
Q2_P37788	C919228.1	Smp_158480	AMP dependent ligase, putative	0.27
Q2_P21035	C804288.1	-	glutathione peroxidase 7 [Homo sapiens]	0.27
Q2_P05882	C810644.1	Smp_046430	ubiquitin-specific peptidase 42 (C19 family)	0.29
Q2_P30711	C905469.1	Smp_105360	Notch	0.29
Q2_P38356	C921416.1	Smp_130170	ubiquitin 1, putative; NEDD8 [Homo sapiens]	0.45
Q2_P35165	C914173.1	Smp_020170	voltage-dependent calcium channel	0.49
Q2_P08644	C902082.1	Smp_181150	ABC transporter, putative	0.52
Q2_P01145	C802678.1	Smp_100390	cullin 3 [Homo sapiens]	0.53
Q2_P07889	C900200.1	Smp_158460	breast cancer anti-estrogen resistance 1 [Homo sapiens]	0.55
Q2_P36177	C915996.1	Smp_043360	nudix-type motif 6 isoform a [Homo sapiens]	0.56
Q2_P36268	C916112.1	Smp_170820	multidrug resistance protein 1, 2, 3, putative	0.57
Q2_P27355	C813427.1	Smp_074160	proteasome maturation protein [Homo sapiens]	0.58
Q2_P31182	C906514.1	Smp_004730	voltage-dependent calcium channel	0.62
Q2_P08701	C902158.1	Smp_020270	voltage-dependent calcium channel	0.63
Q2_P08404	C901707.1	Smp_056440	superoxide dismutase [mn], putative	0.66
Q2_P22162	C805866.1	Smp_049600.x	DNAj (hsp40) homolog, subfamily C, member, putative	0.67
Q2_P22815	C806740.1	Smp_156150	calcium-activated potassium channel	0.67
Q2_P16841	C917848.1	Smp_064380	aspartate aminotrasferase, putative	0.72
Q2_P32712	C909486.1	Smp_003190	26S proteasome subunit P28-related	0.72
Q2_P21772	C805348.1	Smp_168480	apoptosis inhibitor, putative	0.73

**Table 3:** Representative list of 30 induced genes in *S*. *mansoni* adult worms in response to MG-132 treatment.

ProbeName	Contig	Gene Smp	Gene annotation	Fold Change
Q2_P08423	C901740.1	Smp_121780	serine/threonine kinase	1.32
Q2_P37907	C919420.1	Smp_085310.2	26S proteasome regulatory subunit S3, putative	1.40
Q2_P37636	C918731.1	Smp_067890	proteasome subunit alpha 2 (T01 family)	1.49
Q2_P05423	C810048.1	Smp_140260	apoptotic peptidase activating factor 1 isoform b [Homo sapiens]	1.53
Q2_P40330	JAP04182.S	-	proteasome 26S non-ATPase subunit 5 [Homo sapiens]	1.56
Q2_P36517	C916741.1	Smp_017070	26S protease regulatory subunit S10b, putative	1.59
Q2_P24543	C809045.1	Smp_042150	Dynein light chain 1. cytoplasmic, putative	1.68
Q2_P25077	C809737.1	Smp_052870	26S proteasome non-ATPase regulatory subunit, putative	1.83
Q2_P13768	C911179.1	Smp_122680	oligophrenin, putative	1.90
Q2_P30182	C904635.1	Smp_123620	C85 protease (C85 family)	2.63
Q2_P20759	C803931.1	Smp_076740	30S ribosomal protein S8, putative	2.70
Q2_P27440	C900096.1	Smp_126760	proteasome (prosome, macropain) activator subunit 4 [Homo sapiens]	2.71
Q2_P21612	C805121.1	Smp_021750.2	bicoid-interacting protein related	3.60
Q2_P29186	C903263.1	Smp_131070	condensin, putative	4.28
Q2_P02684	C805352.1	-	asp (abnormal spindle)-like, microcephaly associated [Homo sapiens]	4.78
Q2_P01813	C803891.1	Smp_061310.3	chromosome 15 open reading frame 24 [Homo sapiens]	4.83
Q2_P33526	C910959.1	Smp_150140	PI3kinase, putative	4.94
Q2_P23192	C807248.1	Smp_124120	DNA polymerase epsilon subunit b, putative	4.95
Q2_P14061	C911711.1	Smp_139810	Ubiquitin-protein ligase BRE1, putative	5.17
Q2_P28151	C901770.1	Smp_141580	protein kinase	5.42
Q2_P13391	C910365.1	Smp_143920	integrator complex subunit 9 isoform 1 [Homo sapiens]	6.03
Q2_P12516	C908685.1	Smp_045470	homeobox protein prospero/prox-1/ceh-26. putative	6.18
Q2_P29138	C903195.1	Smp_129470	dystroglycan 1 preproprotein [Homo sapiens]	6.22
Q2_P01162	C802712.1	-	discoidin domain receptor family, member 2 precursor [Homo sapiens]	7.33
Q2_P12459	C908573.1	Smp_160830	phospholipase d–related	8.01
Q2_P12817	C909349.1	Smp_097620	vacuolar protein sorting 25 [Homo sapiens]	8.41
Q2_P37055	C917660.1	Smp_164620	cortactin, putative	9.95
Q2_P00261	C800825.1	Smp_065190.2	thioredoxin-like protein, putative	10.8
Q2_P12610	C908920.1	Smp_130590	inhibitor of apoptosis 1, diap1, putative	11.2
Q2_P01503	C803351.1	-	jumonji domain containing 1B [Homo sapiens]	19.5

### Validation of microarray experiments by quantitative real-time PCR

**Fig 3:**
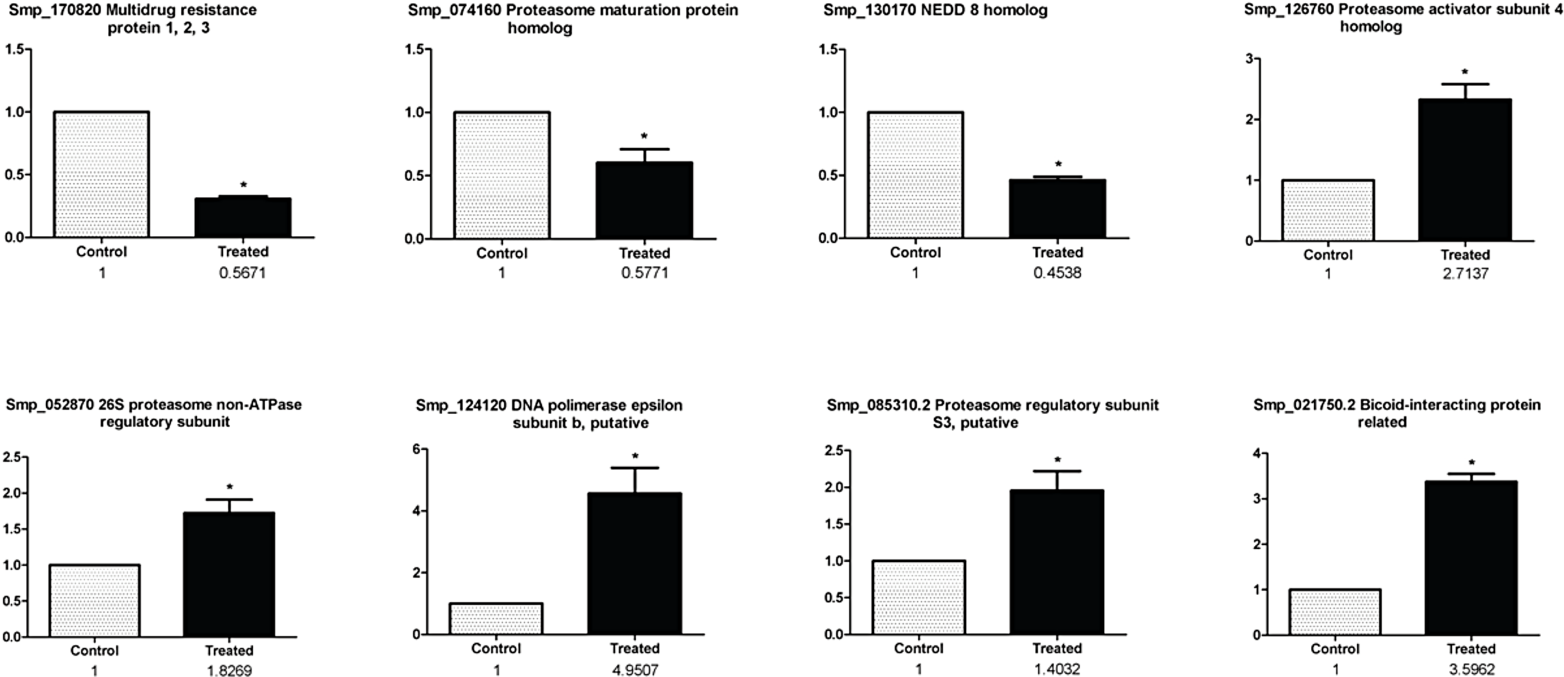
Microarray results validation by real-time PCR. Validation is shown for a group of selected differentially expressed genes in *S*. *mansoni* adult worms treated with MG-132 compared with control parasites. Real time PCR data, expressed as Fold Change (normalized to the control group) are displayed as a bar graph while the corresponding data from the microarray (fold change) are shown below in numbers. The asterisk (*) indicates a statistically significant change (p < 0.05, t-test) when comparing treated with control samples.

Candidate genes were selected for real-time RT-PCR validation, based on their potential role in the parasite biology and the hypothetical connection between the expression changes and the phenotypical effects observed in the adult worms treated with MG-132, as we elaborate in detail in the Discussion section further below. The selected genes are: NEDD8 (Smp_130170); multidrug resistance protein 1, 2, 3 (Smp_170820); Bax regulator of apoptosis (Smp_095190); POMP (Smp_074160); β subunit of DNA polymerase ε (Smp_124120); 26S proteasome non-ATPase regulatory subunit (Smp_052870); subunit 4 activator of the 26S proteasome (Smp_126760); S3 regulatory subunit of the 26S proteasome (Smp_085310.2) and the bicoid protein interaction (Smp_021750.2). Alpha-tubulin was used as reference gene. Fig 3 shows that all selected genes were validated; Fig 3 also shows the results of the comparison between the data obtained by microarray and real-time RT-PCR. The consistency between microarray and real time RT-PCR data was verified through Pearson’s correlation (*r* = 0.98).

### Scanning electron microscopy

**Fig 4:**
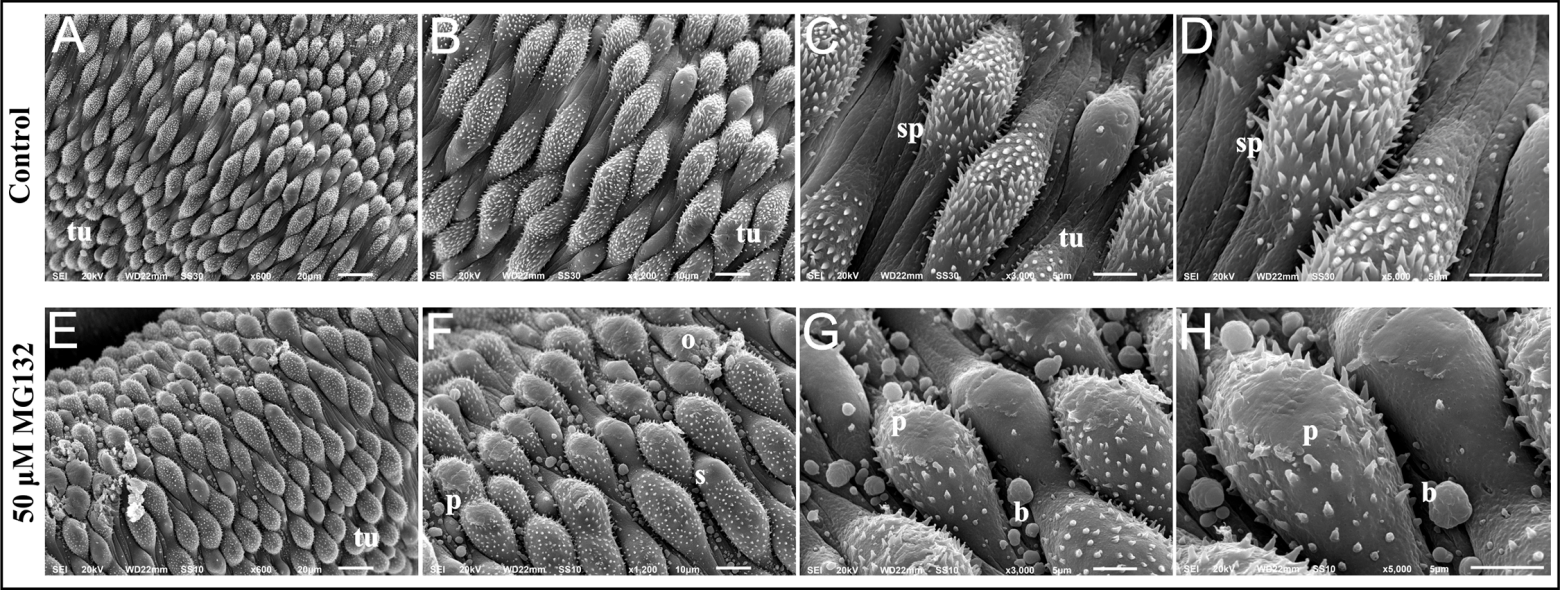
Scanning electron microscopy of the tegument of *S*. *mansoni* adult worms. (A, x600) Normal morphology of adult worm tegument. (B, x1200) and (C, x3000) magnification of normal morphology of *S*. *mansoni* tegument showing the large number of tubercles (tu) and spines (sp). (D, x5000) Magnification of normal morphology of *S*. *mansoni* tegument showing spines (sp). (E, x600) Morphology of *S*. *mansoni* tegument after treatment with 50 μM MG-132 for 24 h. (F, x1200) Magnification showing tegumental changes in treated male adult worms: peeling (p), swelling (s), outbreak (o). (G, x3000) and (H, x5000) Magnification showing peeling (p) and bubbles (b) in the tegument promoted by MG-132.

Parasite tegument was observed through scanning electron microscopy in order to document the damage caused by exposure to 50 μM MG-132 (Fig 4). The analyses were performed in the posterior region of the parasite. Fig 4A to 4D show a normal tegument in *S*. *mansoni* adult worms in the control group, which is rich in well-organized tubercles with many spines randomly distributed throughout the body. These tubercles are surrounded by spines. Fig 4E to 4H show changes in the tegument caused by the exposure to MG-132. Upon treatment with MG-132, peeling (p) was observed. Additionally, it was possible to identify outbreaks (o) and swelling (s) in the tubercles.

## Discussion

Proteasomes are major sites for ubiquitin-targeted protein degradation in eukaryotic cells [52,53] and play fundamental roles in the development of diverse parasites [10,13–17,22]. In *S*. *mansoni*, MG-132 proteasome inhibitor has been shown by our group to cause a marked accumulation of high-molecular weight ubiquitinated proteins, as detected by immunoblotting with anti-ubiquitin antibodies, thus evidencing the on target-engagement of the worm proteasome by MG-132 [22]. In the current study, we measured the large-scale gene expression changes induced in adult worms upon treatment with MG-132, in an attempt to document the molecular events that underlie the phenotypical changes previously reported by Guerra-Sá *et al*. [22], such as the separation of adult worm pairs and interruption of female oviposition caused by MG-132.

When the information about the effect of MG-132 available at the IPA tool database was associated with all *S*. *mansoni* differentially expressed genes (that have human gene homologs), a network was constructed (S2 Fig) and we identified genes (highlighted in Fig 2) that, according to the literature, are affected by MG-132 (changes in expression level, protein activity or localization). It suggests that there are conserved responses related to the ubiquitin-proteasome system in the parasite and in model animals such as humans, mice and rats. In this respect, it should be noted that MG-132 is a well-documented inhibitor of cysteine proteases (cathepsins and calpain) as well as of the proteasome multicatalytic protease [54] in diverse animals. In *P*. *falciparum* for example, MG-132 is a dual-target inhibitor, inhibiting both cysteine proteases and the ubiquitin proteasomal system [55]. We cannot rule out the possibility that these alternate protease targets may influence some of the expression changes detected here.

While many reports in the literature have investigated the role of the proteasome complex in *S*. *mansoni* biology and phenotype [22,56–58], here we assessed for the first time the large-scale differential expression of proteasome genes caused by a proteasome inhibitor; curiously, most of these genes were up-regulated under MG-132 treatment. Thus, we observed the induction of genes encoding catalytic and regulatory particles (α1, α2 and Rpt4) and proteasome activator subunit 4 (an activator PA200 subunit), which might participate in regulation of the proteolytic activity [59]. Our data also showed increased gene expression of 26S proteasome non-ATPase regulatory subunit 5 (S5) (Smp_125800 homologue to Sjp_0022550), which is encoded by the PSMD5 gene [60] and has been identified as a functional homolog of the chaperone Hsm3 in mammals [61]. In this way, our data suggest that under the inhibition caused by MG-132, treated parasites are engaged in the recovery of the proteasome function through the synthesis of new proteasome units. Similarly, De Paula et al. [62] showed that different types of stress could drastically up-regulate the expression of two proteasome genes, namely SmHul5 and SmUbp6, suggesting that the proteasome is important in the cellular stress response in this parasite.

In contrast to the up-regulation of most proteasome genes, we found down-regulation of two chaperone homologs, which are involved with proteasome regulatory particle (RP) base assembly [63], namely chaperone homologs Hsm3 and Nas6, as well as down-regulation of the homolog of the proteasome maturation protein (POMP) gene (Smp_074160), which is among the most highly down-regulated genes (Table 2). In yeast, the POMP gene, also known as *Ump1*, plays a fundamental role in maturation of the 20S proteasome catalytic core [64,65] and deletion of *Ump1* results in accentuated decrease of 26S proteasome catalytic core [66].

Therefore, the present results indicate that while proteasome inhibition by MG-132 activates the expression of genes encoding subunits of the 26S proteasome, it also decreases the expression of genes encoding important 19S chaperones assembly and 20S proteasome maturation protein. These results suggest that the proteolytic activity of the proteasome fails to get recovered, as shown by the accumulation in the parasite of high molecular weight ubiquitinated proteins [22], thus possibly resulting in cellular damage and a decreased viability of the parasites. Repression of NEDD8 (Smp_130170) expression, observed here in the presence of MG-132 (Fig 2), might add to the factors contributing to the loss of parasite viability. NEDD8 encodes an ubiquitin-like protein that plays an important role in cell cycle progression, growth and survival, thus regulating cell growth, viability and development [67]. Attachment of NEDD8 to cullins activates their associated E3 ubiquitin ligase activity, and thus promotes polyubiquitination and proteasomal degradation of cyclins and other regulatory proteins [68]. Besides NEDD8 down-regulation, we observed a significant down-regulation of cullin 3 (Smp_100390) (Table 2), the gene encoding a partner of NEDD8 which appears as an important gene hub in the network of MG-132 affected genes (S2 Fig), thus reinforcing the hypothesis that the proteolytic activity of the proteasome is considerably reduced.

We observed among the down-regulated genes (Table 2), an important number of genes encoding ion channels such as: high voltage-activated calcium channel Cav1 (Smp_020270, Smp_020170 and Smp_004730), calcium-activated potassium channel (Smp_156150) and hyperpolarization activated cyclic nucleotide-gated potassium channel (Smp_153100). The ion channels are pore-forming membrane proteins and protein complexes that underlie electrical excitability and fast neurotransmission, as well as other rapidly-changing biological functions in cells [69]. The decreased expression of these transcripts observed here could explain the previously detected separation of adult worm pairs induced by MG-132 [22], since changes in the membrane potential and ionic homeostasis imbalance might result in impaired muscular contraction in these parasites. Also, tegument damage observed here might be associated with changes in transcript levels of ion channels.

In addition, we observed down-regulation of genes encoding multidrug resistance (MDR) proteins such as ABCB1 (Smp_170820) (Table 2 and Fig 2), ABC transporter (Smp_181150) (Table 2) and copper ABC transporter (Smp_144970) (S1 Dataset). These transporters are responsible for the detoxification of xenobiotics, metal ions and metabolic toxins. Noteworthy, praziquantel has an opposite effect, namely an increased expression of these transporters [35,70] and a reduced sensitivity to praziquantel correlates with higher levels of MDR transporters [70]. Interestingly, genetic knockdown and pharmacological inhibition of the MDR transporters disrupts egg production by *S*. *mansoni* [71]. The reduced expression of MDR genes caused by MG-132 might contribute to the sensitivity of the parasite to the drug, and be related to the previously observed MG-132-induced drastic inhibition of egg production of *S*. *mansoni* adult worms [22]. In this respect, the observed marked down-regulation of CPEB gene (Smp_137460) with MG-132 seems relevant (Table 2); in *Xenopus*, CPEB controls the oocytes germ cells development [72,73], suggesting that germ cells development and cell division, essential to oogenesis, could be inhibited and impaired by the decrease in CPEB expression caused by MG-132 in *S*. *mansoni*. Further direct characterization of oocytes function in the female parasite is required. In addition, it is noteworthy that the present results were obtained with the pool of adult male and female worms and we expect that they will open new perspectives to explore the gender specific effects of MG-132 in future studies.

Intriguingly, while one gene encoding a pro-apoptotic protein, Apoptotic protease activating factor 1 (Apaf-1) (Smp_140260) was up-regulated, a number of homolog genes in the apoptotic pathway were affected in the opposite direction, suggesting a possible diminished apoptosis. Thus, Bax (Smp_095190) encoding a key pro-apoptotic protein was among the top down-regulated genes in *S*. *mansoni* exposed to MG-132 (Table 2), while two genes contributing to apoptosis inhibition were among the top up-regulated (Table 3). One is the gene encoding the inhibitor of apoptosis protein 1 (IAP1) (Smp_130590), which was one of the most highly induced genes in the treated parasites (11-fold up-regulated) (Table 3). Molecular characterization of the inhibitor of apoptosis in *Schistosoma japonicum* (SjIAP) has shown that its transcription occurs predominantly at the developmental stages present in the mammalian final host [74]. The second up-regulated gene (Smp_139810) encodes the ubiquitin ligase BRE1 (Table 3); this protein exhibits an anti-apoptotic activity in *Sacharomyces cerevisiae*, and an increased level of Bre1p in *S*. *cerevisiae* has an evident role in protecting from hydrogen peroxide-induced cell death, whereas its deletion enhances cell death [75]. Our results suggest that up-regulation of BRE1 in *S*. *mansoni*, as a result of MG-132 treatment, may contribute to dampen the activation of the cell death processes.

Presence of the mitochondrial Bcl-2-regulated apoptosis pathway in *Schistosoma* [76] makes this pathway evolutionarily close to that of humans and *Caenorhabditis elegans*. In mammalian cells, Bcl-2 family proteins such as Bax are central regulators of apoptosis, which is a process essential for cellular homeostasis, life development, and prevention of pathological conditions [77]. Together, our results suggest a deregulated apoptosis pathway due to a decreased expression of its key-activating component Bax, and an increased expression of apoptosis inhibitors IAP1 and BRE1. This result is in contrast to the induction pattern seen in mammalian tumor cells treated with MG-132 [78,79] where an increased apoptosis [78] and an increased expression of genes such as p38 kinase and JNK1 [79] have been observed. In fact, in line with the opposite effect of MG-132 on the parasite compared with tumor cells, the *S*. *mansoni* p38 kinase (Smp_133020) and JNK1 (Smp_172240) gene homologs were detected here as expressed, however not significantly affected by MG-132, thus reinforcing the finding that apoptosis might not be induced, but rather diminished in the treated parasite. This might not be surprising, because in tumor cells, treatment with proteasome inhibitors prevents NF-κB activation and leads to toxic accumulation of misfolded proteins, which in turn activates JNK1 and apoptosis [80], whereas *S*. *mansoni* does not have an NF-κB homolog. Overall, deregulation of the apoptosis pathway caused by MG-132 might compromise *S*. *mansoni* cell homeostasis, and consequently decrease parasite viability.

## Conclusions

The proteasome inhibition in *S*. *mansoni* led to changes in expression of genes involved in cellular processes other than protein degradation, which documents that the proteasome is essential for gene expression regulation, and suggests that the proteasome might be an important molecular target for the design of new drugs against this parasite.

## Supporting information

S1 Fig *In vitro* effect of MG-132 on the viability of *S*. *mansoni* adult worms. (TIF)

S2 Fig Interaction network between MG-132 and gene products. All interactions were previously described in the literature in model organisms, according to the Ingenuity Pathway Analysis (IPA) tool as detailed in the Methods. Yellow lines show the interactions between MG-132 and the *S*. *mansoni* gene homologs that were detected as differentially expressed, and blue lines show the interactions among these differentially expressed genes.
(JPG)

S1 Table List of oligonucleotides used in real time PCR to validate the microarray data. (PDF)

S2 Table Names of the corresponding *S*. *mansoni* homolog genes shown in the network of Fig 2, and summary of the effect of MG-132 on these genes in model organisms, as described in the literature. (XLSX)

S1 Dataset List of repressed genes in MG-132 treated adult worms (*S*. *mansoni*). (XLSX)

S2 Dataset List of induced genes in MG-132 treated adult worms (*S*. *mansoni*). (XLSX)
