# Supplementary figures and images for: Effects of proteasome inhibitor MG-132 on the parasite Schistosoma mansoni
Source: PLoS One. 2017 Sep 12;12(9):e0184192. doi: 10.1371/journal.pone.0184192 (PMC5595316; doi:10.1371/journal.pone.0184192)

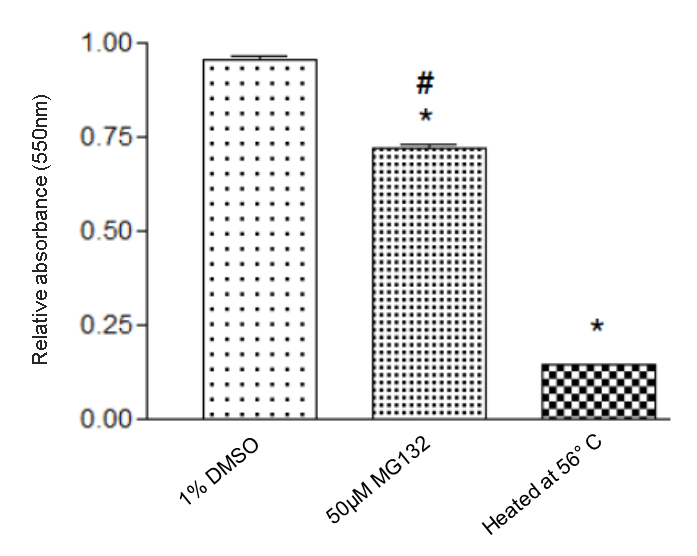

Supplement: S1 Fig — (TIF) [file pone.0184192.s001.tif]

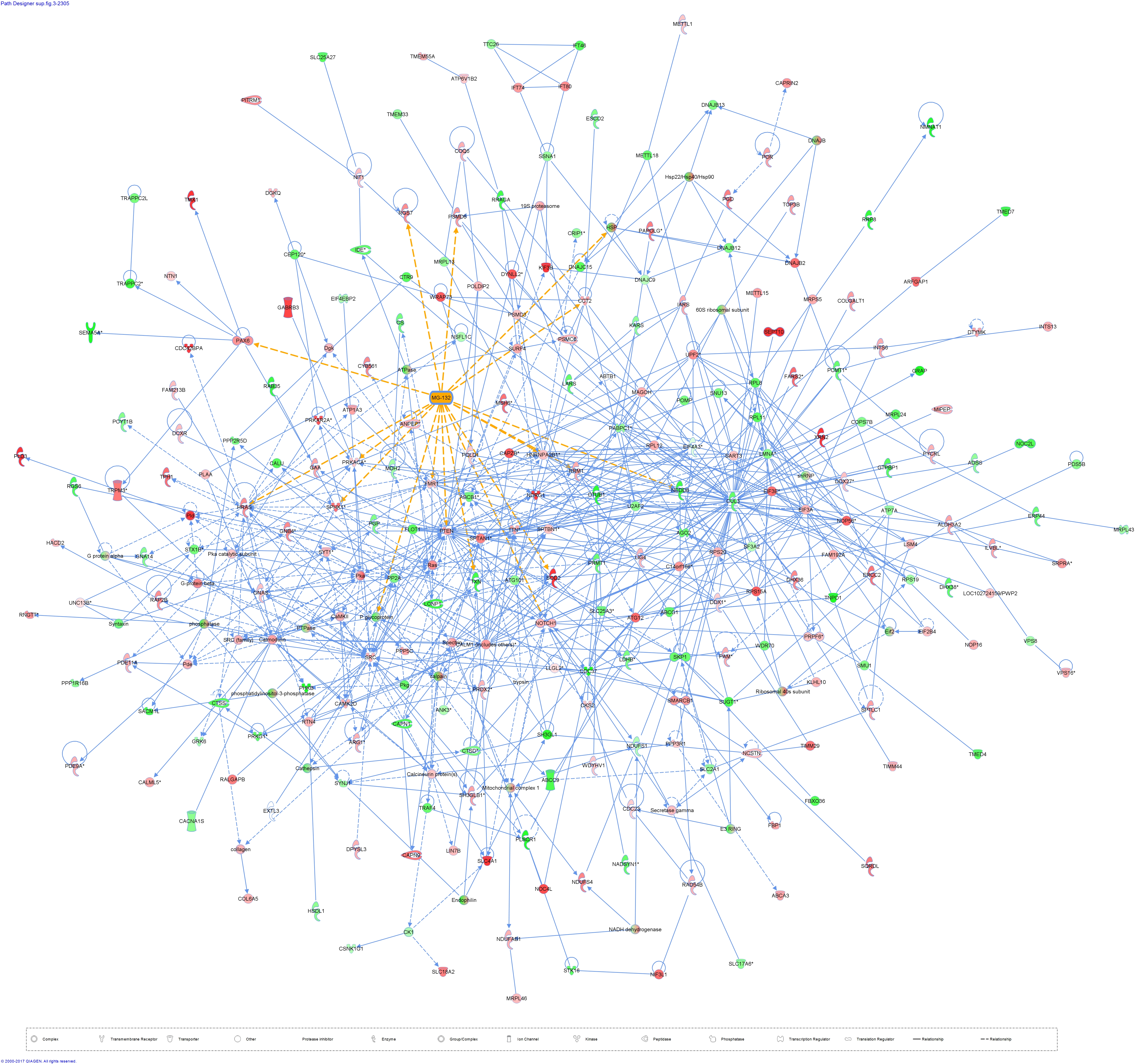

Supplement: S2 Fig — All interactions were previously described in the literature in model organisms, according to the Ingenuity Pathway Analysis (IPA) tool as detailed in the Methods. Yellow lines show the interactions between MG-132 and the S. mansoni gene homologs that were detected as differentially expressed, and blue lines show the interactions among these differentially expressed genes. (JPG) [file pone.0184192.s002.jpg]
