# Supplementary material for: Effects of proteasome inhibitor MG-132 on the parasite Schistosoma mansoni
Source: PLoS One. 2017 Sep 12;12(9):e0184192. doi: 10.1371/journal.pone.0184192 (PMC5595316; doi:10.1371/journal.pone.0184192)
Supplement: S1 Table — (PDF) [file pone.0184192.s003.pdf]

S1 Table. List of oligonucleotides used in real time PCR to validate the microarray data.

Gene name	Primer pair sequence (5′-3′)	
	Forward	Reverse
Smp_130170	TGGAACCAGATGAAATCACG	GGTGAAAAAGGTGTGGAACG
Smp_170820	ACAGTCACTTGGACGTTCTGC	TCCCCACGTTAACTGGAATG
Smp_074160	GGAAGATTCTGAAACCGTTGG	TCAATTCGAGAAAACCAATTGTC
Smp_124120	TGACAACTACTCAGGCTGGTTC	GATCCAAAGTATCTGGCCTATCC
Smp_052870	TGCCAGGCTTTCGATTAGAC	CCTTCTTCAATTAGGCTGTTCG
Smp_126760	AGGGCAAGGTCAATCTGATG	TGTACGCCATGATGTATGCTG
Smp_085310.2	TGCCGGAAAGCTTAACACTC	TTGCATGCCTCTTCTAGGTG
Smp_021750.2	TCCTAGGCCTAATGAACCATTC	GAATGGATGCCGGAGTTG
M80214*	GAAATGCTTGTTGGGAGTTG	TTATCACTTGGCATCTGTCC

* Alpha-tubulin, constitutive gene
